# T-cell–predominant inflammation in livedoid vasculopathy and response to baricitinib

**DOI:** 10.1016/j.jdin.2025.10.019

**Published:** 2025-12-05

**Authors:** Dan-dan Wei, Zhuo-xue Nong, Dan Huang, Shu-fen Wang, Kun-qian Huang, Si-jian Wen

**Affiliations:** a Department of Dermatology and Venereology, The First Affiliated Hospital of Guangxi Medical University, Nanning, Guangxi, China; b Department of Dermatology and Venereology, Wuming Affiliated Hospital of Guangxi Medical University, Nanning, Guangxi, China

**Keywords:** baricitinib, immunohistochemistry, inflammation, livedoid vasculopathy, pathological feature

*To the Editor:* Livedoid vasculopathy (LV) is typically associated with coagulation abnormalities, for which anticoagulation therapy is the standard treatment. However, recent studies suggest that inflammation may also contribute to LV's pathogenesis.^1^ This study aims to investigate the role of inflammatory processes in LV.

We conducted a retrospective analysis of clinical data from 36 LV patients. Additionally, tissue samples were obtained from 10 LV patients (consecutive cases diagnosed at our institution between July 2023 and January 2024) and 3 control patients with leukocytoclastic vasculitis for immunohistochemical analysis. The expression of CD3, CD4, CD8, CD20, Langerhans protein, STAT6, myeloperoxidase (MPO), and myelin basic protein was evaluated (Supplementary Material 1, available via Mendeley at https://data.mendeley.com/datasets/p663cmn8vn/1).

Pathological examination revealed lymphocytic infiltration in all 36 LV patients. Fifteen patients showed epidermal damage, characterized by neutrophil and erythrocyte infiltration, leading to blister formation. Immunohistochemical analysis revealed that CD3, CD4, CD8, MPO, and STAT6 were positive in all 10 LV patients and 3 leukocytoclastic vasculitis patients, while myelin basic protein was negative in all cases. CD20 was positive in 20% of LV patients, and Langerhans protein was positive in 40%. In the 3 leukocytoclastic vasculitis patients, CD20 was negative in all cases, and Langerhans protein was positive in 2 patients (Supplementary Material 2, available via Mendeley at https://data.mendeley.com/datasets/p663cmn8vn/1).

While previous studies have reported minimal lymphocytic infiltration in LV tissues^2^; our study observed significant lymphocytic infiltration, fibrinoid degeneration, and hyaline thrombus formation in dermal vessels. Immunohistochemical analysis consistently showed positivity for CD3, CD4, CD8, and MPO, with CD20 remaining negative. CD3, CD4, and CD8 are primarily expressed on T lymphocytes, while CD20 is a B-cell marker, and MPO, expressed in neutrophils and monocytes, plays a key role in inflammation.^3^ These findings highlight the central role of T lymphocyte infiltration in LV's pathogenesis.

Building on these results, we initiated a single-arm, open-label clinical trial to evaluate the safety and efficacy of baricitinib in treating LV. Ten new LV patients were treated with an initial dose of either 2 mg/day (*n* = 3) or 4 mg/day (*n* = 7), based on disease severity. Baseline and 3-month follow-up data were collected.

Among the 10 patients treated, 4 were male and 6 female, with a mean age of 30.5 years. After 1 month, 8 patients showed improvements in bruising, ulcer healing, and pain reduction, while 2 patients showed no significant improvement. At 3 months, 8 patients exhibited significant clinical improvement (*P* < .05), as detailed in Table I, Supplementary Material 1, available via Mendeley at https://data.mendeley.com/datasets/p663cmn8vn/1 and Fig 1.

**Table I:** Demographic and clinical scores at baseline and after 3 months of treatment

Sex	Age (y)	Ethnicity	Disease duration (mo)	Duration of follow-up (wk)	LV composite clinical score (BL/3M)			DLQI (BL/3M)	NRS (BL/3M)
					Erythema	Ulcer	Pain		
F	39	Asian	240	12	2/0	3/1	2/1	19/5	6/1
F	39	Asian	24	10	2/0	2/0	2/0	16/0	6/0
F	27	Asian	168	12	1/2	0/0	3/3	11/13	8/8
F	39	Asian	96	11	2/3	2/1	1/0	15/3	3/0
F	24	Asian	24	10	2/0	0/0	0/0	10/10	0/0
F	22	Asian	28	10	2/0	2/0	2/1	16/3	6/1
M	28	Asian	36	10	1/0	1/0	1/0	10/0	3/0
M	24	Asian	11	10	2/0	1/0	2/1	13/3	6/1
M	30	Asian	12	10	1/0	0/0	2/1	11/2	6/1
M	33	Asian	4	9	3/0	1/0	2/1	18/3	6/1

The LV Composite Clinical Score was adopted from the comprehensive clinical scoring system first established and applied by Weishaupt et al (2016) to assess disease severity and activity in patients with LV. This scoring system evaluates 3 major clinical manifestations characteristic of LV—erythema, ulceration, and physician-assessed pain—with a total score ranging from 0 to 9. In this study, erythema and ulceration scores were jointly assessed by 2 board-certified dermatologists, while pain scores were self-reported by patients using a visual analog scale (VAS). Specific evaluation criteria are as follows:

Erythema: 0 (none); 1 (mild); 2 (moderate); 3 (severe).

Pain: 0 (none); 1 (mild); 2 (moderate); 3 (severe).

Ulceration: 0 (none); 1 (epidermal); 2 (dermal); 3 (subcutaneous tissue).

*3M*, After treatment for 3 months; *BL*, baseline; *DLQI*, Dermatology Life Quality Index; *F*, female; *M*, male; *NRS*, numeric rating scale.

**Fig 1:**
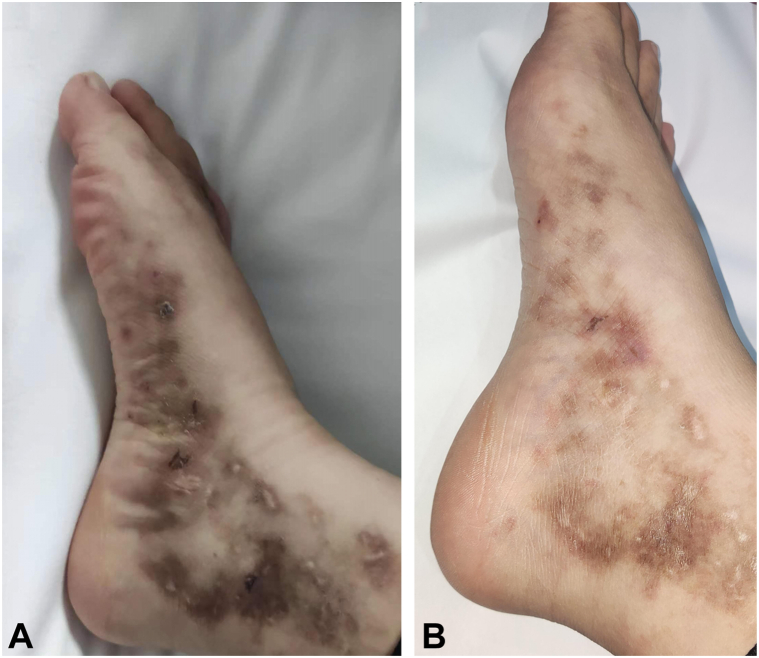
Skin lesion images of a patient diagnosed with LV before and 4 months after baricitinib treatment. **A,** Before baricitinib treatment: Multiple erythema, ulcers, crusting, some chronic white atrophic scars, and localized pigmentation observed on both feet dorsum and medial/lateral malleolar regions. **B,** Four months after baricitinib treatment: Erythema significantly diminished, ulcers healed, with residual *white atrophic scars* and some pigmentation.

Our study found that 80% of patients treated with baricitinib showed significant improvement. However, 2 patients (on the 2 mg dose) exhibited minimal changes, partly due to interruptions in therapy. Recent literature suggests that Janus kinase inhibitors may be effective in treating LV,^4^^,^^5^ further supporting the role of inflammation in its pathophysiology.

In conclusion, our study highlights the critical role of inflammation in LV's pathogenesis. Baricitinib appears to be an effective and safe treatment for refractory LV and may offer an alternative therapeutic option. However, this study has limitations, including the lack of a standard treatment control group and a small sample size. Larger-scale prospective studies are needed to validate these findings.

## Conflicts of interest

None disclosed.
